# Expired Patents

**Published:** 1906-08

**Authors:** 


### EXPIRED PATENTS.

July 2nd, 1906.

406349, Dental chair, D. D. Hepburn, London, England.

The above list of patents is furnished by Davis & Davis, solicitors of American and foreign patents, Washington, D. C., and St. Paul Building, New York City.

